# Supplementary material for: Ionic Liquid Transdermal Patches of Two Active Ingredients Based on Semi-Ionic Hydrogen Bonding for Rheumatoid Arthritis Treatment
Source: Pharmaceutics. 2024 Apr 1;16(4):480. doi: 10.3390/pharmaceutics16040480 (PMC11053956; doi:10.3390/pharmaceutics16040480)
Supplement: Supplementary file 1 [file pharmaceutics-16-00480-s001.zip › pharmaceutics-2908671-File S1.pdf]

1. Western blotting（The following groups are Control, Model, KAT, AAT, AKAT）

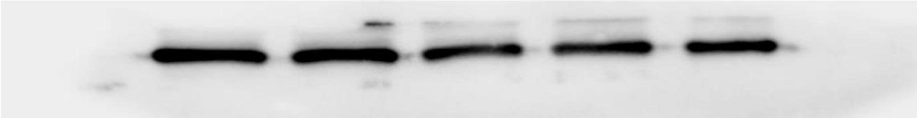

This is the blots image of the experimental group for JAK2.

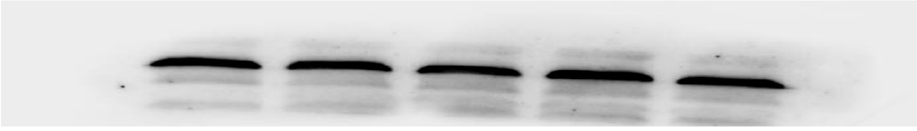

This is the blots image of the experimental group for STAT3.

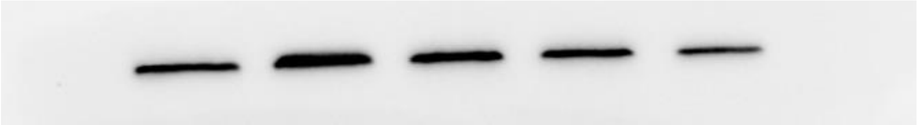

This is the blots image of the experimental group for P-STAT3.

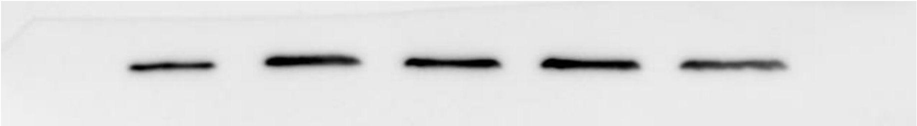

This is the blots image of the experimental group for IL-6.

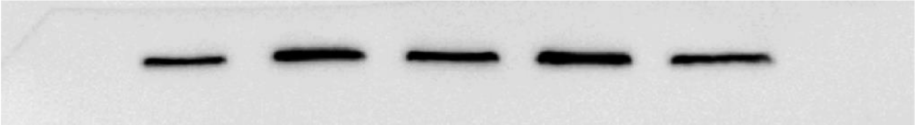

This is the blots image of the experimental group for TNF-α.

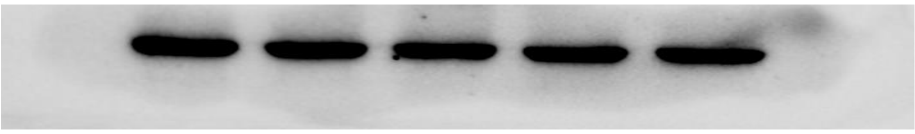

This is the blots image of the experimental group for β-actin.
