# Supplementary material for: Ionic Liquid Transdermal Patches of Two Active Ingredients Based on Semi-Ionic Hydrogen Bonding for Rheumatoid Arthritis Treatment
Source: Pharmaceutics. 2024 Apr 1;16(4):480. doi: 10.3390/pharmaceutics16040480 (PMC11053956; doi:10.3390/pharmaceutics16040480)
Supplement: Supplementary file 1 [file pharmaceutics-16-00480-s001.zip › Supplementary Material.pdf]

Supplementary Material

Faxing Zhang †, Lu Li †, Xinyuan Zhang, Hongyu Yang, Yingzhen Fan, Jian Zhang, Ting Fang, Yaming Liu, Zhihao Nie and Dongkai Wang *

Department of Pharmaceutics, School of Pharmacy, Shenyang Pharmaceutical University, No. 103, Wenhua Road, Shenyang 110016, China; zhangfx199901@163.com (F.Z.)

* Correspondence: wangycsyphu@126.com

† These authors contributed equally to this work.

Supplementary Methods

1. HPLC method

C18 column (200 mm × 4.6 mm, 5 μm) was used for the se-paration of KTP and ATR. The mobile phase was a mixture of methanol andacetic acid solution (0.5%, v/v) at 70:30 (v/v) and 30:70 (v/v), then adjusted to pH 4.0 using triethylamine. The flow rate was 1 mL/min and the wavelength of the detector was 248 nm for the determination of both drugs.

2. *In vitro* percutaneous permeability assay

Cumulative 24-hour drug permeability per unit area:

$$Q_i = (C_i \times V + \Sigma_{i=1}^{n-1} C_{i-1} \times V_i) \,/\, A \qquad \text{(S1)}$$

Q24h is 24 h cumulative drug permeability per unit area, C_i and C_{i-1} are the concentrations of i and i-1 samples. times the concentration of the sample, V_i is the sampling volume (2.0 mL), V is the diffusion cell volume (4.0 mL)

3. Calculation of foot swelling degree in rats:

$$\text{(Swelling degree)}\ S\% = \frac{V_t - V_n}{V_n} \times 100\% \qquad \text{(S2)}$$

where Vn and Vt are the circumferences of the toes measured before and after injection of complete Freund's adjuvant.

4. Swelling severity score requirements

RA severity was determined visually based on an inflammatory score on a scale from 0 to 4, where 0 = no erythema and swelling; 1 = erythema and mild swelling limited to the tarsals or ankle joint; 2 = erythema and mild swelling extending from the ankle to the tarsals; 3 = erythema and moderate swelling extending from the ankle to the metatarsal joints; and 4 = erythema and severe swelling encompassing the ankle, foot and digits, or limb ankylosis. Changes in paw thickness in rats during drug treatment (0day, 12day, 24day), it was observed that paw thickness in the drug-treated group gradually tended to favor the control group.

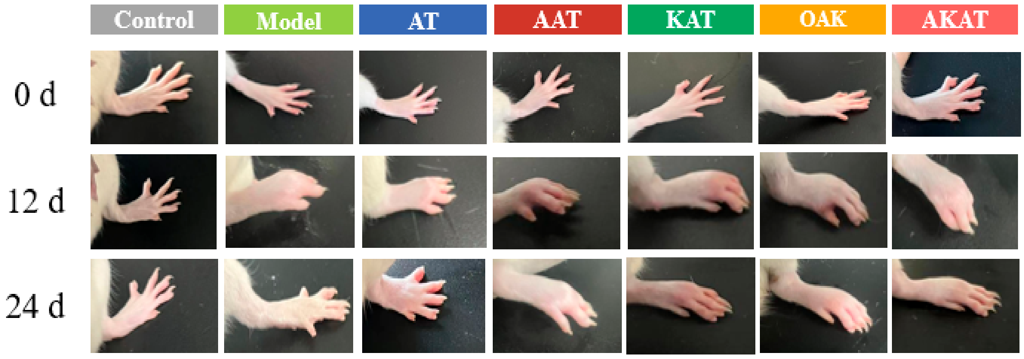

Figure S1. Rats' paw thickness changes during HIHDM-PSA treatment.

5. Beam balance test scoring requirements

The scoring criteria were as follows: score 0, all four limbs were on the board in a balanced state; score 1, one limb was able to grasp the wood or rock on the wood; score 2, one or two limbs slipped off the wood; score 3, three legs slipped off the wood; and score 4, hung on the wood and fell after struggling.

6. Skin irritation experiment:

$$EI = \frac{EI_{24h}}{EI_0} \tag{S3}$$

Initial erythema values were measured as EI_0, Then, the patches were removed after 24 h, and EI_{24h} values were determined.

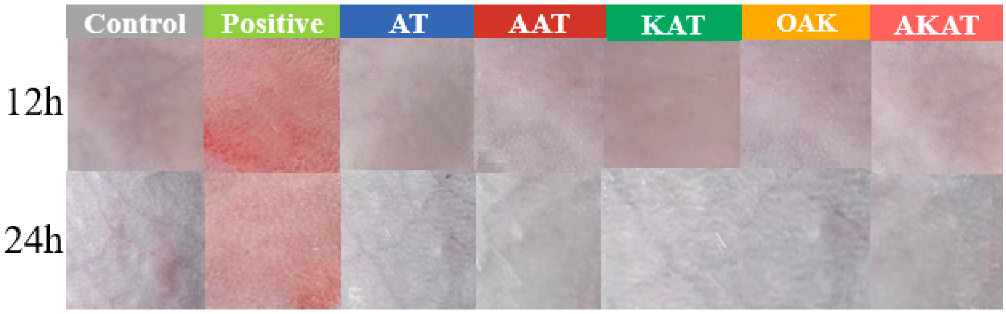

Figure S2. Schematic representation of skin irritation at 12h and 24h in the in vitro erythema assay.
